# Nucleation experiments on a titanium-carbon system imply nonclassical formation of presolar grains

**DOI:** 10.1126/sciadv.add8295

**Published:** 2023-01-13

**Authors:** Yuki Kimura, Kyoko K. Tanaka, Yuko Inatomi, Coskun Aktas, Jürgen Blum

**Affiliations:** ^1^ Institute of Low Temperature Science, Hokkaido University, Kita-19, Nishi-8, Kita-ku, Sapporo 060-0819, Japan.; ^2^ Astronomical Institute, Tohoku University, 6-3 Aoba, Aoba-ku, Sendai 985-8578, Japan.; ^3^ Institute of Space and Astronautical Science, Japan Aerospace Exploration Agency, 3-1-1 Yoshinodai, Chuo-ku, Sagamihara, Kanagawa 252-5210, Japan.; ^4^ School of Physical Sciences, SOKENDAI (Graduate University for Advanced Studies), 3-1-1 Yoshinodai, Chuo-ku, Sagamihara, Kanagawa 252-5210, Japan.; ^5^ Institut für Geophysik und Extraterrestrische Physik, Technische Universität Braunschweig, Mendelssohnstr. 3, D-38106 Braunschweig, Germany.

## Abstract

Just as the shapes of snowflakes provide us with information on the temperature and humidity of the upper atmosphere, the characteristics of presolar grains in meteorites place limits on their formation environment in a stellar outflow. However, even in the case of well-characterized presolar grains consisting of a titanium carbide core and a graphitic carbon mantle, it is not possible to delimit their formation environment. Here, we have demonstrated the formation of core-mantle grains in gravitational and microgravity environments and have found that core-mantle grains are formed by a nonclassical nucleation pathway involving the three steps: (i) primary nucleation of carbon at a substantially high supersaturation, (ii) heterogeneous condensation of titanium carbide on the carbon, and (iii) fusion of nuclei. We argue that the characteristics of not only core-mantle grains but also other presolar and solar grains might be accurately explained by considering a nonclassical nucleation pathway.

## Teaser

Microgravity experiments suggest that presolar TiC core–graphitic mantle grains are formed by three-step nonclassical processes.

## INTRODUCTION

Fine cosmic dust grains are ubiquitously distributed in the interstellar environment. Most of these grains were initially produced in the gas outflows of evolved stars. Determination of the characteristics of these grains is crucial to the understanding of various astronomical phenomena because circumstellar grains are accelerated in the outflow velocity due to radiation pressure (*1*), contribute to the energy balance by absorbing ultraviolet (high-energy) light and then reemitting infrared (low-energy) light in the interstellar environment (*2*, *3*), provide surfaces for the adsorption and formation of molecules in molecular clouds (*4*, *5*), and become the building blocks of planetary systems (*6*). The contributions of grains to these processes depend on their size, number density, and composition, in addition to environmental parameters such as temperature and pressure. Consequently, attempts have been made to determine the characteristics of solid grains in various astronomical environments by using grain-formation models in conjunction with astronomical observations.

Examples of actual grains that are older than the Solar System can be found in primitive meteorites. These grains, known as presolar grains, show an isotopic signature that links them to a stellar origin (*7*–*9*). Graphitic grains containing carbide nanoparticles are well-known examples of such presolar grains (*10*–*14*). A representative example of such grains consists of a titanium carbide (TiC) core and a graphitic mantle several micrometers in diameter (*10*). Roughly 30% of the graphitic grains contain metallic carbides; these consist of titanium (Ti) [1 to 95 at % (at %) (excluding carbon)], zirconium (0 to 80 at %), molybdenum (0 to 38 at %), and/or ruthenium (0 to 30 at %); about 40% of these are located at the centers of the grains (*13*). In some cases, graphitic grains contain two or more TiC grains (*14*) or even several hundreds of TiC grains (*12*). The TiC grains can vary in size from several tens to several hundreds of nanometers (*14*). Oxygen isotopic anomalies in TiC suggest that the core-mantle grains originated in the gaseous outflows of asymptotic giant-branch (AGB) stars (*13*) or in carbon-rich ejecta, such as the carbon-helium (C-He) zones of core-collapse type II supernovae (*12*).

The structure of the core-mantle grains appears to suggest that the TiC grains were formed initially, and these subsequently became coated with a thick layer of carbon in more distant regions of the stellar outflow. On the basis of this condensation sequence and the sizes of the core and mantle, numerous attempts have been made to characterize various aspects of the formation environment of the grains, such as the gas outflow velocity, the carbon-to-oxygen ratio, the gas density, and the temperature; however, reasonable conditions for grain formation have yet to be identified (*15*–*19*). Some circumstellar dust condenses under conditions far from thermodynamic equilibrium (*20*). Particularly in the case of the first-condensing refractory dust, the onset of condensation in a cooling flow occurs at several hundreds of degrees Kelvin below the nominal condensation limit in thermodynamic equilibrium (*21*–*23*). The degree of supercooling strongly depends on the details of the reaction kinetics, especially for TiC, and the relative order of C and TiC condensation in stellar outflows is unclear. Condensation temperatures obtained from thermodynamic equilibrium calculations can be misleading in nonequilibrium situations.

Condensation sequences can be calculated by means of nucleation theory (*24*–*26*). Unfortunately, however, some missing physical quantities for TiC and C, especially their sticking probability and surface tension on the nanoscale, coupled with a poor understanding of the nucleation pathway have hampered reliable modeling. Here, we report the determination of these missing physical quantities based on a modified classical nucleation theory (MCNT) (*27*), together with the results of our studies on the formation processes of TiC/C grains by modeling the production of core-mantle grains in homogeneous nucleation experiments with analogous particles in our laboratory and in a microgravity environment provided by a sounding rocket (see Materials and Methods and fig. S1). As a first approach and to facilitate experimental treatment, this paper considers environments with a low hydrogen contribution, such as carbon-rich ejecta of core-collapse type II supernovae (*19*, *28*) or possibly carbon-rich Wolf-Rayet stars (*29*).

## RESULTS

**Fig. 1.**
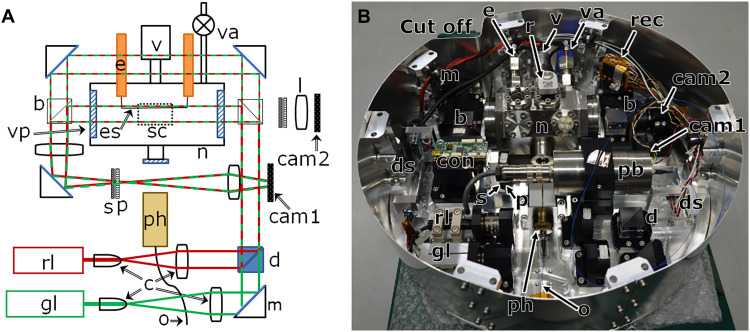
Interferometer and nucleation chamber for the microgravity experiment in the sounding rocket. (**A**) Optics and laser path of the double-wavelength Mach-Zehnder–type laser interferometer and the nucleation chamber (*n*), which are same equipment with that in (*23*). The red and green lines show the optical paths of the red and green lasers (rl and gl), respectively. The interference fringes and real images were recorded by charge-coupled device cameras (cam1 and cam2, respectively) and recorders. The evaporation source and the sample collector are shown as black solid (es) and dotted (sc) lines, respectively. The other labels are as follows: b, beam splitter; c, collimator; d, dichroic mirror; e, electrode; l, lens; m, mirror; o, optical fiber; p, polarizer; ph, pyrometer head; s, short-pass filter; sc, sample collector; v, vacuum gauge; va, valve with a gas line; vp, viewport. (**B**) Photograph of the experimental system. All optics and the chamber were located on a 405-mm-diameter base plate. “Cut off” is a duct for cable connections between the payload and the rocket. The labels are as follows: con, controller for the sample collector; ds, D-sub connectors; pb, pyrometer body; rec, image recorder.

Hot vapors of Ti, C, and TiC were generated by resistive heating of appropriate sources containing the corresponding bulk material under argon (Ar) as a buffer gas introduced into the nucleation chamber to decrease the mean free path, thereby permitting a reduction in the size of the nucleation chamber to a laboratory scale [(*30*); see also Materials and Methods). The nanometer-sized particles that formed through nucleation in the cooling gas mimicked grain formation in the gas ejecta of evolved stars (*22*, *23*, *30*). The entire process involving nucleation and formation of the resulting particles was observed in situ using a double-wavelength Mach-Zehnder–type laser interferometer (Fig. 1) and a transmission electron microscope (TEM), respectively, as detailed in Materials and Methods.

**Fig. 2. F2:**
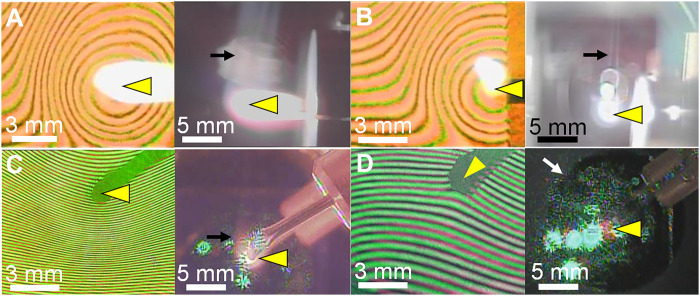
Snapshots of in situ observations of nucleation of dust analogs from the vapor phase. The left and right panels show double-wavelength interference fringes and real images, respectively. The yellow triangles indicate the evaporation source. The smaller deviation of the interference fringes away from the evaporation source roughly corresponds to a temperature gradient. (**A** to **C**) Nucleation of Ti, C, and TiC, respectively, in gravity. The black arrows show smoke following convection currents generated by the corresponding hot evaporation source. (**D**) Nucleation of Ti in microgravity. The white arrow indicates the nucleation front. The nucleation fronts for the gravitational experiments are beneath and near the corresponding evaporation sources (<1 mm) and cannot, therefore, be recognized in these real images. Time series of snapshots for each experiment are shown in figs. S2 to S5.

In all the experiments, changes in the interference fringes occurred simultaneously with heating of the evaporation source due to a decrease in the optical pathlength caused by a decrease in the number density of the Ar gas (see figs. S2 to S5). Once evaporation of the starting material was initiated, the deviation of the interference fringes was compensated by the TiC vapor, which has a higher refractive index than that of Ar. In both gravity and microgravity environments, nucleated particles became evident at high source temperatures in the form of the smoke visible in the real images in Fig. 2. The time that it takes for an evaporated vapor to cool and nucleate depends on the temperature of the evaporation source and the distance between the nucleation position and the evaporation source, which is shortened by convection. Therefore, the time until smoke was generated was 1.4 × 10^−4^ to 1.8 × 10^−3^ s in the laboratory but was 1.1 × 10^−2^ s in the microgravity environment. In nucleation from a supersaturated gas, the effect of gravity on individual atoms or molecules is negligible, whereas its effect on gas convection as a collective motion is substantial, as described below.

### Advantages of microgravity experiments

Under terrestrial gravity, heating an evaporation source generates a macroscopic difference in density that causes convection currents due to the presence of concentration and temperature gradients in the gaseous atmosphere. As a result, the gas densities above and below the evaporation source differ, creating a large inhomogeneity in the nucleation environment. Because cold Ar gas always emerges from below the evaporation source, the temperature gradient is steeper at the bottom of the evaporation source than above it (fig. S6). Because evaporation occurs concentrically, the supersaturation ratio is greatest below the evaporation source, and nucleation occurs predominantly in that region (*31*). The resulting smoke particles rise, carried by a thermal convective flow of Ar.

Under microgravity, both the temperature field and the concentration field of the evaporated gas are arranged concentrically around the evaporation source, and nucleation occurs under uniform conditions. This provides several advantages for the purposes of research (*22*, *30*, *32*). First, like the physical treatment of systems with identical Reynolds numbers in fluid mechanics, the formation process of cosmic dust can be realistically simulated if the cooling time scale, τ_T_, and the collision frequency, υ, are similar (Table 1) (*33*, *34*). In our microgravity experiment, control of the mean free path permitted a simulation of the nucleation process of cosmic grains in the gas outflow from supernovae and AGB stars (*30*). As a result, we were able to measure the sticking probability and surface tension at temperatures similar to the formation temperature of circumstellar grains. Because both these physical quantities depend on the temperature, similarity of the nucleation conditions is important for achieving a realistic simulation of dust growth. Second, we avoided any enhancement of nucleation due to the generation of turbulence. (Turbulence can cause nucleation, as evidenced by the fact that shaking supercooled water can initiate ice formation.) Third, due to the homogeneity of the nucleation environment, it was possible to minimize the width of the size distribution of the resulting particles, thereby minimizing errors in physical quantities associated with the effects of particle sizes.

**Table 1.** Physical quantities used in the calculations of the condensation process. The constants in the equilibrium vapor pressure were obtained from (*57*) and (*58*) for Ti and TiC, respectively, and by fitting the data in (*58*) for C.

	Gravity	*C*_1_	*C*_2_	*m* (kg)	ρ_m_ (kg m^−3^)	τ_T_ (s)	υ (s^−1^)	*T*_0_ (K)	*D* (m^2^ s^−1^)	*X* (m)
Ti	1	6.358	22,747	7.95 × 10^–26^	4,500	1.8 × 10^–3^	1.8 × 10^6^	2,500	7.12 × 10^–4^	1.1 × 10^–3^
Ti	0	6.358	22,747	7.95× 10^–26^	4,500	2.2 × 10^–2^	1.5 × 10^6^	2,500	1.33 × 10^–3^	5.5 × 10^–3^
C	1	10.609	41,523	2.00 × 10^–26^	2,500	1.4 × 10^–4^	2.8 × 10^7^	3,400	2.69 × 10^–3^	7.5 × 10^–3^
TiC	1	7.652	33,600	9.95 × 10^–26^	4,900	2.8 × 10^–4^	1.1× 10^6^	3,000	1.80 × 10^–3^	7.2 × 10^–3^

### Results of gravitational experiments

Despite their various disadvantages, experiments under terrestrial gravity conditions in the laboratory can still provide us with useful results. From the deviations between the interference fringes of green and red lasers before heating of the evaporation source and the time of nucleation, the nucleation temperature and partial pressure were determined to be 1149 ± 23 K and $176_{-61}^{+91}$ Pa for Ti (Fig. 2A and fig. S2), 2495 ± 50 K and $2766_{-1196}^{+2044}$ Pa for C (Fig. 2B and fig. S3), and 1744 ± 35 K and $465_{-175}^{+265}$ Pa for TiC (Fig. 2C and fig. S4), respectively. The supersaturation ratios for nucleation (*P*/*P*_e_, where *P*_e_ is the equilibrium vapor pressure of the corresponding material) were 1.4 × 10^11^ for Ti, 2.9 × 10^4^ for C, and 1.4 × 10^9^ for TiC. The equilibrium vapor pressure of a material is given by the expression log *P*_M_ (atm) = *C*_1_ – *C*_2_/*T*; the values of constants *C*_1_ and *C*_2_ are given in Table 1. The uncertainty in the equilibrium vapor pressure caused by the decomposition of TiC was confirmed by a molecular dynamics simulation (fig. S7). These results show that an extremely large supersaturation is required for homogeneous nucleation.

**Fig. 3.**
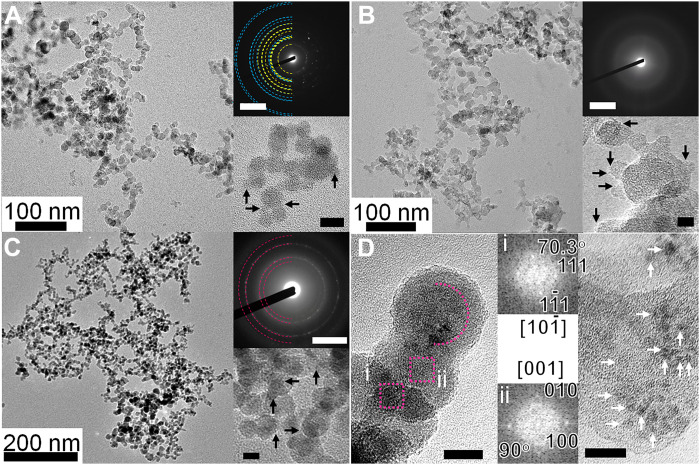
Bright-field TEM images and the corresponding ED patterns of the particles produced in the experiments shown in Fig. 2. [Fig F2]The scale bars in the ED pattern and the TEM images without labels are 5 nm^−1^ and 10 nm, respectively. The arrows show the smallest particles identified in each image. (**A**) Ti particles formed at 1*g*. The ED rings acquired from the corresponding image shown as cyan and yellow semicircles correspond to Ti (Joint Committee on Powder Diffraction Standards (JCPDS), #5-0682) and TiO_2_ (anatase, JCPDS, #21-1272), respectively. The original Ti particles were oxidized by exposure to air during their transfer from the nucleation chamber to the TEM. (**B**) C particles formed at 1*g*. The ED pattern shows a typical amorphous grain. (**C**) TiC particles formed at 1*g*. The ED rings shown as magenta semicircles correspond to the (111), (200), (220) and (311) planes of TiC (JCPDS, #32-1383) from inside to outside. (**D**) TiC core–C mantle particles produced in microgravity. The dotted circle shows a core particle. The fast Fourier transform diffractions of regions i and ii show the formation of TiC crystals. The right-hand image shows tiny grains embedded in a C particle.

The particles produced under gravity were collected directly on a thin film of amorphous carbon supported by a TEM grid, a microgrid, or an amorphous silicon nitride film. These grids were loaded into a TEM and the crystalline structure, size, and size distribution of the particles were determined. The electron diffraction (ED) patterns indicated that the collected particles consisted of Ti, amorphous carbon, and TiC (Fig. 3, A to C, respectively). The Ti nanoparticles in Fig. 3A were oxidized when they were removed from the nucleation chamber and during their transfer to the TEM. Their ED pattern shows the presence of titanium oxide (TiO_2_), mainly in the form of anatase, in addition to pure Ti. The size of the Ti nanoparticles increased by 90% as a result of oxidation. The diameters of the resulting particles were determined by manual measurements on individual spherical particles, excluding those cases in which two particles overlapped or were connected by a thick neck. The measured average diameters were 27, 12, and 11 nm for Ti, C, and TiC, respectively (fig. S8). Many fine particles were also present, as shown by the arrows in the enlarged images in Fig. 3. The diameters of these fine Ti, C, and TiC particles were 2.5, 1.6, and 0.8 nm, respectively. The size of the original Ti particles before oxidation was estimated to be ~1.3 nm. Carbon and TiC particles did not oxidize and change in size during the experimental procedures because negligible amounts of oxidation occur at ambient temperatures and pressures.

### Determination of physical quantities

The experimental conditions of nucleation temperature, time scale for cooling, and particle size should have a direct relationship to the physical properties according to nucleation theory (*27*). In particular, the sticking probability and the surface tension are the physical properties that produce the greatest uncertainties in calculations based on nucleation theory. From our work, these two physical properties can be determined using the MCNT based on the experimental conditions (*22*, *23*). To compensate for the disadvantage of classical nucleation theory, the MCNT contains a correction term such that the energy difference between the two phases (Δ*G*) is zero when the nucleus size is a monomer (*35*).

**Fig. 4.**
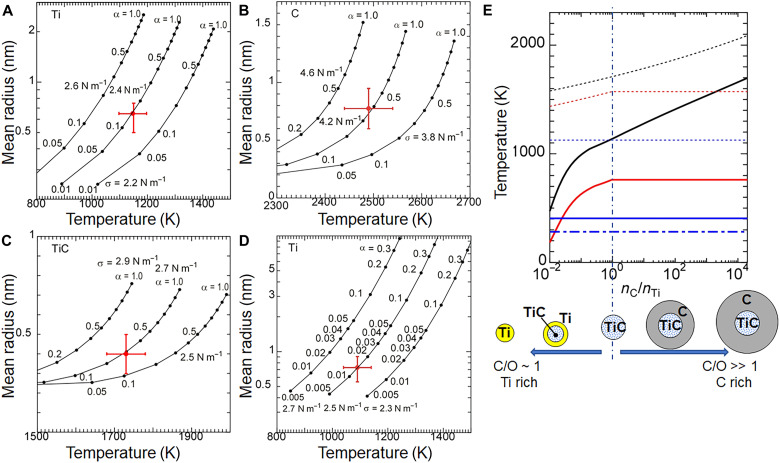
Determination of sticking probabilities, α, and surface tensions, σ, at various nucleation temperatures and mean particle radii by using the MCNT model. (**A**) Ti particles formed in 1*g*. (**B**) C particles formed at 1*g*. (**C**) TiC particles formed in 1*g*. (**D**) Ti particles formed in microgravity. The error bars in (A) to (D) represent the errors in the nucleation temperature for the horizontal axis and the mean radius of fine particles for the vertical axis. (**E**) Nucleation temperature in a gas outflow from the type II supernova using values of α and σ obtained from the gravitational experiment (solid lines) in (A) to (C) and the microgravity experiment (chain line) in (D) in terms of the C/Ti ratio, which depends on the C/O ratio, with a cooling time of 1 year. Dotted lines show the equilibrium temperature for Ti (blue), C (black), and TiC (red) in terms of the C/Ti ratio. The number density of Ti (*n*_Ti_ = 9.2 × 10^10^ m^−3^) was fixed based on type II supernovae (*19*) and the gaseous number density [≍3.6 × 10^17^ m^−3^: (*47*)]; therefore, the size of the resulting particles increases as the C/Ti ratio increases. The number density of C (*n*_C_ = 1.7 × 10^15^ m^−3^) in the C-He zone of type II supernovae corresponds to right-hand end of the horizontal axis. The vertical dash-dotted line corresponds to C/Ti = 1.

Although attempts were made to determine the physical quantities using the mean size of the resulting particles, no realistic values could be obtained. On the other hand, an analysis using the size of the fine particles gave reasonable values. Figure 4 (A to C) shows the values of the sticking probability (α) and the surface tension (σ) that permit an explanation of the nucleation temperatures and sizes of fine particles in each experiment under gravity conditions, as summarized in Table 2. For all three materials, the sticking probabilities were less than 50%, in contrast to the assumption of a 100% sticking probability that is required in many theoretical models of grain formation to explain the abundances of grains. The surface tension of Ti is somewhat larger than the maximum value of 2.1 N m^−1^ for liquid Ti (*36*), and the surface tension of TiC is between the reported values of 1.58 N m^−1^ for (011) and 3.53 N m^−1^ for (110) at room temperature as obtained by density functional theory calculations (*37*). In the case of carbon, the surface tension varies markedly, from 0.04 to 4.8 N m^−1^, depending on the crystalline structure and the crystal face (*38*). For astronomical estimations of grain formation, values of about 1.40 N m^−1^ have typically been used (*39*). However, our experimental results show that the surface tension can be as large as 4.2 ± 0.2 N m^−1^ under the supersaturation conditions where carbon grains are nucleated. The surface tension of nanoparticles is known to differ from that of the corresponding bulk material (*21*, *40*). Our results of a smaller sticking probability and a larger surface tension indicate that grain formation through homogeneous nucleation is more difficult than is commonly believed, and they imply the formation of many tiny particles.

**Table 2.** Sticking probabilities and surface tensions.

	Gravity	Sticking probability	Surface tension (N m^−1^)
Ti	1	0.15 ± 0.10	2.4 ± 0.1
Ti	0	0.014 ± 0.010	2.5 ± 0.1
C	1	0.39 ± 0.15	4.2 ± 0.2
TiC	1	0.29 ± 0.10	2.7 ± 0.2

### Secondary growth

How do circumstellar particles with sizes of the order of 100 nm, as suggested by astronomical observations and the presence of micrometer-sized presolar grains in meteorites, form? Because nanoparticles that contact one another readily fuse to decrease their total surface energy, the final size of the resulting particles might be increased by secondary collisional growth (*31*, *41*, *42*). This phenomenon is known to occur in the nucleation process and is referred to as particle-mediated nucleation (*43*). This is one of the several nonclassical nucleation processes that have attracted much attention in recent years, and it has been reported to occur in various systems, suggesting that it might be a universal phenomenon (*44*, *45*). We therefore assume that the fine particles are the original particles formed from a single nucleus.

Ti, C, and TiC grains with diameters of 27, 11, and 12 nm, respectively, can be formed by the fusion of 7000, 420, and 2600 fine particles, respectively. The collision frequency among fine particles was 10^6^ to 10^8^ s^−1^ under our experimental conditions (*42*), suggesting that each particle could be formed within 0.01 s. Because the convection velocity of Ar gas (*46*) is 10 to 15 cm s^−1^, grains must grow to their final size while moving ~1 mm after nucleation about 1 mm below the evaporation source. At this stage, the particles pass through a region with a relatively high temperature of about 1000 K (fig. S6), which is favorable for fusion growth. This theory is based on the assumption that when particles collide with each other, they always fuse to form a spherical particle; even if the probability of fusion is an order of magnitude smaller, the theory would still be consistent with the experimental results. The fluffy structures shown in Fig. 3 are the result of collisions after cooling in which particles did not fuse with each other, although some particles became partially fused and formed a thick neck.

### Results of microgravity experiments and determination of physical properties

Our microgravity experiment was performed onboard the MASER 14 sounding rocket of the Swedish Space Corporation, launched at 8:52 a.m. CEST (Central European Summer Time) on 24 June 2019, from the Esrange Space Center, Sweden. As expected, concentric smoke was visible because of the absence of thermal convection in the microgravity environment (Fig. 2D and fig. S5). The smoke expanded gradually and produced particles that were deposited on an amorphous silicon nitride film of a TEM grid. Because the evaporation source, a Ti wire wound around a C rod, was electrically heated, the partial pressure of Ti in the mixed vapor of Ti and C was relatively high and, therefore, particle formation began with homogeneous nucleation of Ti. Nevertheless, TiC nanocrystals with a carbon mantle did form (Fig. 3D). Nucleation occurred 5.5 mm from the evaporation source at 1079 ± 22 K with a supersaturation ratio of 3.5 × 10^12^ (*P*_Ti_ = 178 Pa). The distance from the evaporation source to the nucleation position was quite far compared with the value of 1.1 mm for the Ti experiment in the laboratory, where nucleation occurred at 1149 K. This is a result of the weaker temperature gradient near the evaporation source in the microgravity environment. As in the terrestrial experiments, fine particles with a diameter of 1.5 nm initially formed (Fig. 3D) and then fused to produce larger grains. The average diameter of the resulting particles was 23 nm, which requires 3900 fine particles. The sticking probability of Ti in the microgravity experiment was determined from the size of primary particles, as in the terrestrial experiments, and its value was 0.014 ± 0.010 (Table 2), which is an order of magnitude smaller than the corresponding value obtained under terrestrial gravity (0.15 ± 0.10). This large difference might be caused by an enhancement of nucleation due to turbulence generated by convection currents in the terrestrial experiments. Turbulence produces regions where the hot evaporated gas and cold argon gas mix, generating localized fluctuations in temperature and density. Nucleation is then enhanced due to the presence of localized regions of high supersaturation. In other words, under microgravity conditions, nucleation is suppressed and, therefore, the measured sticking probability tends to have a smaller value than that determined under conditions of terrestrial gravity. Therefore, the true sticking probability can only be determined in a microgravity environment (Table 2). Consequently, the sticking probabilities of C and TiC obtained in microgravity experiments is much smaller than the values listed in Table 2 that were obtained in a gravitational environment.

## DISCUSSION

Contamination can cause heterogeneous nucleation with concomitant effects on experimental results, such as an increased sticking probability. Regardless of whether contaminants act as substrates for heterogeneous nucleation or not, no contamination was identified during the experimental process or analyses, including the TEM observations. Because contamination can promote nucleation and, thereby, increase the sticking probability, a low sticking probability indicates that the amount of contamination is very small. In this experiment, the apparatus was evacuated and then filled with high-purity Ar gas, and the valve was closed. The laboratory experiments were then performed immediately, whereas the microgravity experiment was performed 16 hours later. In both cases, air contamination due to leakage was below the detection limit (~100 Pa). Even 1 month later, the pressure change in the gas-filled experimental apparatus was of the same order as that caused by changes in room temperature. Therefore, the larger sticking probability in the ground experiment was not due to a greater degree of contamination than that in the microgravity experiment.

On the basis of terrestrial experiments, the nucleation temperatures of Ti, C, and TiC in a gas outflow from the type II supernovae (C-He zone) were numerically calculated in terms of the ratio of C and Ti, with a cooling time of 1 year (Fig. 4E). The number density of the Ti vapor was fixed at a value of 9.2 × 10^10^ m^−3^, based on the adopted atomic zone of a 25-solar-mass supernova (*19*) and the gaseous number density [≈3.6 × 10^17^ m^−3^; (*47*)]. In all cases where the C/Ti ratio was less than ~1, the nucleation temperatures of Ti, C, and TiC were each below the equilibrium temperature, i.e., the temperature at which the flux of molecules evaporating from the surface exactly balances the flux of molecules condensing on the surface for the three components. This suggests that once nucleation occurs, the nuclei grow by continuous deposition of Ti and C atoms. In the case of C/Ti ratios of 1 to 2000, C nucleates homogeneously at first. Then, Ti atoms begin to deposit on the C nuclei, and TiC forms on the C nucleus because the equilibrium temperature of C and TiC is higher than the nucleation temperature of C. Because the size of a critical nucleus of C is only several atoms due to the very large supersaturation (only five atoms for the experiment in Fig. 2B), the carbon atoms in the particle core are no longer identifiable as such in the final grain after the deposition of additional material. Although the equilibrium temperature of Ti is lower than the nucleation temperature of C, Ti should be locked into TiC due to the abundance of C. Note how the lower sticking probability of Ti in the microgravity environment decreased the nucleation temperature of Ti in Fig. 4E. Similarly, the actual nucleation temperatures of C and TiC should be much lower than the solid lines in Fig. 4E. Then, the condition for the formation of C particles with Ti expands to C/Ti ratios larger than 2000. The overview remains the same regardless of whether the Ti number density is 50 or 10% lower than the values discussed above (fig. S9), suggesting that the formation process should also be valid in a gas outflow that differs somewhat from that of a 25-solar-mass supernova. Thus, tiny C nuclei are initially formed through homogeneous nucleation under supersaturated conditions in a gas outflow from an evolved star. Ti then deposits on the C nuclei together with C to form C particles containing TiC. Thousands of fine particles then fuse to produce a larger particle through particle-mediated growth (*43*–*45*). The TiC in the primary C particles might transition to a lower energy TiC core–C mantle state through diffusion of Ti during the particle-mediated growth. Assuming a bulk diffusion coefficient of 1.5 × 10^−20^ m^−2^ s^−1^ at 1079 K [(*48*); see Materials and Methods], it would take only 2.2 years for diffusion of TiC in a core–mantle grain of representative size (1 μm). The required time scale is comparable to that (<2000 days) for dust formation in a model (*28*). Diffusion would be accelerated by the increased diffusion coefficient inherent in nanoparticles (*49*), the latent heat of condensation, and radiation heating from the star. A supernova graphitic grain containing hundreds of TiC nanocrystals (*12*) might be in an intermediate form in our proposed mechanism. Although TiC core–C mantle particles were formed in our experiments (Fig. 3D), obtaining direct evidence for this diffusion process is the subject of our next challenge. If the sticking probability of C does not decrease substantially in the static conditions of a microgravity environment, to explain the formation of TiC core–C mantle grains, the C/Ti ratios need to be smaller than that in the C-He zone of the gas outflow from type II supernovae. Finding the upper limit of the C/Ti ratio at which the nucleation temperature of carbon is lower than the equilibrium temperature of TiC, by determination of the sticking probability of C, is also among our future challenges.

A knowledge of the nucleation pathway and physical properties of nanoscale materials is essential for estimating the amounts of grains formed in various astronomical objects, including asymptotic giant stars, planetary nebulae, and planetary atmospheres, in addition to supernovae ejecta. Furthermore, such knowledge is useful in controlling the formation of nanoparticles in industrial dry processes that may be environmentally more friendly than chemical processes because of no effluent. It will also provide a direction for controlling nanoparticle formation in a wide range of research fields related to materials science.

## MATERIALS AND METHODS

### Experimental equipment and analytical method

Hot vapors of C and/or Ti were generated by resistive heating of the corresponding bulk material in an Ar gas atmosphere at a pressure of ~40,000 Pa (fig. S10); the Ar reduces the mean free path of the evaporated vapor, thereby permitting a reduction in the physical size of the nucleation chamber. Nanometer-sized particles formed in the gas outflow from the evaporation source as the gas cooled. Condensation occurred through homogeneous nucleation, as there was no substrate near the evaporation source.

The nucleation processes were directly observed by using a double-wavelength Mach-Zehnder–type laser interferometer (Fig. 1); this permitted the detection of differences in the refractive index of less than one part per million. These differences correspond to changes of several tens of nanometers in the optical path length. The refractive index of the nucleation environment in the gas phase can be described as a function of the temperature, *T* (K), and partial pressure of the evaporated material, *P*_M_, at each laser wavelength as follows

$$N_{M(T,P)}-1=\frac{\left[N_{M(273.15,P_{0})}-1\right]}{1+a\Delta T}\frac{P_{M}}{P_{0}}$$ (1)

where *a* is the coefficient of volume expansion (0.003663 K^−1^ for Ar and 0.003661 K^−1^ for the evaporated materials in this experiment), Δ*T* = *T* – 273.15 K, and pressure *P*_0_ = 101325 Pa. The values of the refractive indices of Ar (*N*_Ar_), Ti (*N*_Ti_), TiC (*N*_TiC_), and C (*N*_C_) at 10^5^ Pa and 293.15 K that we used in our analysis were as follows: (*N*_Ar_ – 1) = 2.813 × 10^−5^ at 532 nm and 2.792 × 10^−5^ at 635 nm (*50*–*53*), (*N*_Ti_ – 1) = 7.023 × 10^−4^ at 532 nm and 8.086 × 10^−4^ at 635 nm (*54*), (*N*_TiC_ – 1) = 10.777 × 10^−4^ at 532 nm and 11.346 × 10^−4^ at 635 nm (*55*), and (*N*_C_ – 1) = 4.045 × 10^−4^ at 532 nm and 4.399 × 10^−4^ at 635 nm, respectively *56*). To obtain reference temperatures, platinum resistance thermometers (Pt100, type MC-0805; Netsushin Co. Ltd., Saitama) were attached to copper electrodes that were connected to the evaporation source and to the sample collector located ~30 mm from the evaporation source in the chamber. The temperature of the evaporation source and the total pressure in the nucleation chamber were measured with a two-color pyrometer (ISQ5-LO/MB25, Yamari Industries Ltd., Yokohama) and a pressure gauge (HAV-60KP-V, SENSEZ Corporation, Tokyo), respectively.

The product of the interference fringe shift, Δ*d*, and the laser wavelength, λ, is proportional to the change in the optical path length, *L*, defined as *L* = *Nl*, where *l* is the physical length of the evaporation source (in this experiment, ~70 mm). The shift in the positions of the interference fringes of the green laser (Δ*d*_G_) and red laser (Δ*d*_R_) after heating are given by the expressions

$$\Delta d_{G}=[N_{G,\mathrm{Ar}(T_{i},P_{i})}-N_{G,\mathrm{Ar}(T,P-P_{M})}-N_{G,M(T,P_{M})}+1]\frac{l}{\lambda_{G}}$$ (2)

and

$$\Delta d_{R}=[N_{R,\mathrm{Ar}(T_{i},P_{i})}-N_{R,\mathrm{Ar}(T,P-P_{M})}-N_{R,M(T,P_{M})}+1]\frac{l}{\lambda_{R}}$$ (3)

respectively, where *T*_i_ and *P*_i_ are the initial temperature and pressure of Ar before the source temperature was elevated, and the subscripts G and R indicate quantities for the green and red lasers, respectively. Because the total pressure in the chamber, *P*, was monitored by a pressure gauge and Δ*d*_G_ and Δ*d*_R_ could be observed in the image, *T* and *P*_M_ could be determined simultaneously by simple calculations using Eqs. 1 to 3. The deviations of the fringe shift at the nucleation front just before nucleation are shown in figs. S2 to S5. The supersaturation ratio at the moment of nucleation could then be determined.

### Microgravity experiment in a sounding rocket

The basic configuration of the equipment for the microgravity experiment was the same as that for the terrestrial experiment. Nucleation experiments with Ti, C, and TiC in a microgravity environment were performed on board the sounding rocket MASER 14 of the Swedish Space Corporation, launched from the Esrange Space Center, Sweden, on 24 June 2019. Countdown began at 23:05 local time on 23 June 2019, and the rocket was launched at 08:52 on 24 June 2019. Microgravity was experienced 66 s after launch (*Y* + 66) and ended at *Y* + 448 with a microgravity duration of 382 s (fig. S1). The maximum altitude attained was 244.7 km at *Y* + 257, and the spacecraft spun up to 152° per second after the end of microgravity at *Y* + 448 for reentry. A parachute for deceleration was deployed at *Y* + 584, and the main parachute was deployed at *Y* + 610. The maximum gravitational acceleration during deceleration was 11.5*g*. Communication with the sounding rocket was lost at *Y* + 842. The landing point of the payload was 74.6 km from the launch site, and the payload returned at 12:05 CEST on the launch day. The launch proceeded successfully as planned.

### Particle analysis

The collected particles were examined by TEM (JEM-2100F, JEOL Ltd., Tokyo) with an acceleration voltage of 200 kV at Hokkaido University, Japan, to determine their crystalline structure, particle size, and size distribution.

### Methods for determining the sticking probability and surface tension

We performed numerical calculations, based on nucleation theory, for nonequilibrium condensation from a cooling gas containing the appropriate material (Ti, C, or TiC) (*27*). We compared our calculations with our experimental results to obtain the sticking probability and the free energies for the formation of molecular clusters, which determine the nucleation rate. The number density of the monomer gas *n*_1_(*t*) is given by

$$n_{1}(t)=n_{1}(0)-\int_{0}^{t}J(t^{\prime }){\left[\frac{r(t,t^{\prime })}{r_{1}}\right]}^{3}dt^{\prime }$$ (4)

where *J*(*t*′) is the nucleation rate at time *t*′, and *r*(*t, t*′) is the radius of clusters nucleated at *t′* and measured at *t*. The growth equation of a radius of clusters is expressed as

$$\frac{\partial r(t,t^{\prime })}{\partial t}=\alpha \frac{4\pi }{3}r_{1}^{3}n_{1}(t)v_{\mathrm{th}}$$ (5)

The radius of the critical nuclei can be expressed as $r(t^{\prime },t^{\prime })=i_{*}^{1/3}r_{1}$, where *i*_∗_ is the number of atoms in a critical cluster, i.e., the smallest thermodynamically stable cluster. In the calculations, we consider a gaseous system that cools with a characteristic time τ_T_. In this case, the temperature *T* of the gas as a function of time *t* is given by *T*(*t*) = *T*_0_ exp(−*t*/τ_T_), where the initial temperature *T*_0_ corresponds to the temperature of the heated evaporation source in the experiments. The nucleation rate *J* is given by

$$J={\left\{\sum_{i=1}^{\infty }\frac{1}{R^{+}(i)n_{e}(i)}\right\}}^{-1}$$ (6)

where *n*_e_(*i*) is the equilibrium number density of *i*-mers (clusters containing *i* atoms) and *R*^+^(*i*) is the accretion rate from an *i*-mer to an (*i* + 1)-mer

$$R^{+}(i)=\alpha n_{1}v_{\mathrm{th}}(4\pi r_{1}^{2}i^{\frac{2}{3}})$$ (7)

where α is the sticking probability, *v*_th_ $\left[=\sqrt{kT/(2\pi m)}\right]$ is the thermal velocity of the gas, and the radius of a molecule *r*_1_ is defined as (3 *m/*4πρ_m_)^1*/*3^, where *m* is the mass of a molecule and ρ_m_ is the bulk density. The equilibrium number density of *i*-mers *n*_e_(*i*) is given by

$$n_{e}(i)=\frac{P}{kT}\exp\left(-\frac{\Delta G_{i}}{kT}\right)$$ (8)

where *P* is the partial pressure for the gas to condense, *k* is the Boltzmann constant, and ∆*G_i_* is the free energy associated with the formation of a cluster of size *i* from the gas phase. For ∆*G_i_*, we apply the MCNT formula, in which the formula from classical nucleation theory is modified by adding an extra term to the free energy for cluster formation to satisfy the condition that the free energy of the monomer is zero (*34*). The resulting expression is as follows

$$\Delta G_{i}=-(i-1)kT\;\ln\;S+\sigma A_{1}(i^{\frac{2}{3}}-1)$$ (9)

where *S* (= *P*/*P_e_*) is the supersaturation ratio and *A*_1_ (=$4\pi r_{1}^{2}$) is the surface area of a monomer. Although σ corresponds to the surface tension in the MCNT, we treat it as a fitting parameter to explain our experimental results. By using Eqs. 4 to 9, we modeled the condensation process. The time scale for cooling τ_T_ was taken as the time required for the gaseous Ti, C, or TiC to arrive at the nucleation site by diffusion from the evaporation source: τ_T_ ≈ *X*^2^*D*^−1^, where *X* is the distance from the evaporation source to the nucleation site, and *D* is the diffusion coefficient. In the estimation, we use the diffusion coefficient given by *D* = *v*_mean_ λ/3, where *v*_mean_ is the mean velocity of the gas with the mean temperature between the evaporation source and the nucleation site and λ is the mean free path of a gas molecule, given by ${\left(\sqrt{2}\pi r_{1}^{2}n\right)}^{-1}$, where *n* is the number density of total gas from the total gas pressure measured in the experiment. Values of the physical quantities ρ_m_, *T*_0_, τ_T_, *X*, and *D* are listed in Table 1. Because all the physical quantities except α and σ were known, we could obtain the values of α and σ by fitting the condensation temperature and average particle radius obtained from the experiments.

### Diffusion coefficient of TiC

The concentration and temperature dependencies of bulk diffusion coefficient (*D*) for TiC can be represented by the expression

$$D=D_{0}\exp[\beta (1-B)]\exp\left(-\frac{Q}{RT}\right)$$ (10)

where β is a concentration-dependent parameter, *B* is the carbon-titanium ratio, *Q* is the activation energy, and *R* is the universal gas constant. If the carbon-titanium ratio is assumed to be unity, and the values of *D*_0_, *Q*, and *T* are 5.75 × 10^−6^ m^2^ s^−1^, 3.0 × 10^5^ J mol^−1^, and 1079 K, respectively (*48*), then *D* = 1.5 × 10^−20^ m^2^ s^−1^. In the case of representative sizes of the core-mantle grains (1 μm) and TiC particles (10 nm) obtained in this experiment, the diffusion times (=*r*^2^/*D*) are estimated to be 2.2 years and 1.9 hours, respectively; 2.2 years is a reasonable period for the time scale of grain formation in the gas ejecta of a supernova. On the other hand, 1.9 hours is too long compared with the time scale of the experiment (~1 s). TiC particles obtained in this experiment, like that shown in Fig. 3D, probably resulted from an enhancement of diffusion by the latent heat of condensation and from the increased diffusion coefficient inherent in nanoparticles (*49*).

## Supplementary Materials

### This PDF file includes:

Figs. S1 to S10 References
