# Supplementary material for: Nucleation experiments on a titanium-carbon system imply nonclassical formation of presolar grains
Source: Sci Adv. 2023 Jan 13;9(2):eadd8295. doi: 10.1126/sciadv.add8295 (PMC9839320; doi:10.1126/sciadv.add8295)
Supplement: Supplementary file 1 — Figs. S1 to S10 References [file sciadv.add8295_sm.pdf]

Science Advances
AAAS

Supplementary Materials for

Nucleation experiments on a titanium-carbon system imply nonclassical formation of presolar grains

Yuki Kimura *et al.*

Corresponding author: Yuki Kimura, ykimura@lowtem.hokudai.ac.jp

Sci. Adv. **9**, eadd8295 (2023)
DOI: 10.1126/sciadv.add8295

This PDF file includes:

Figs. S1 to S10
References

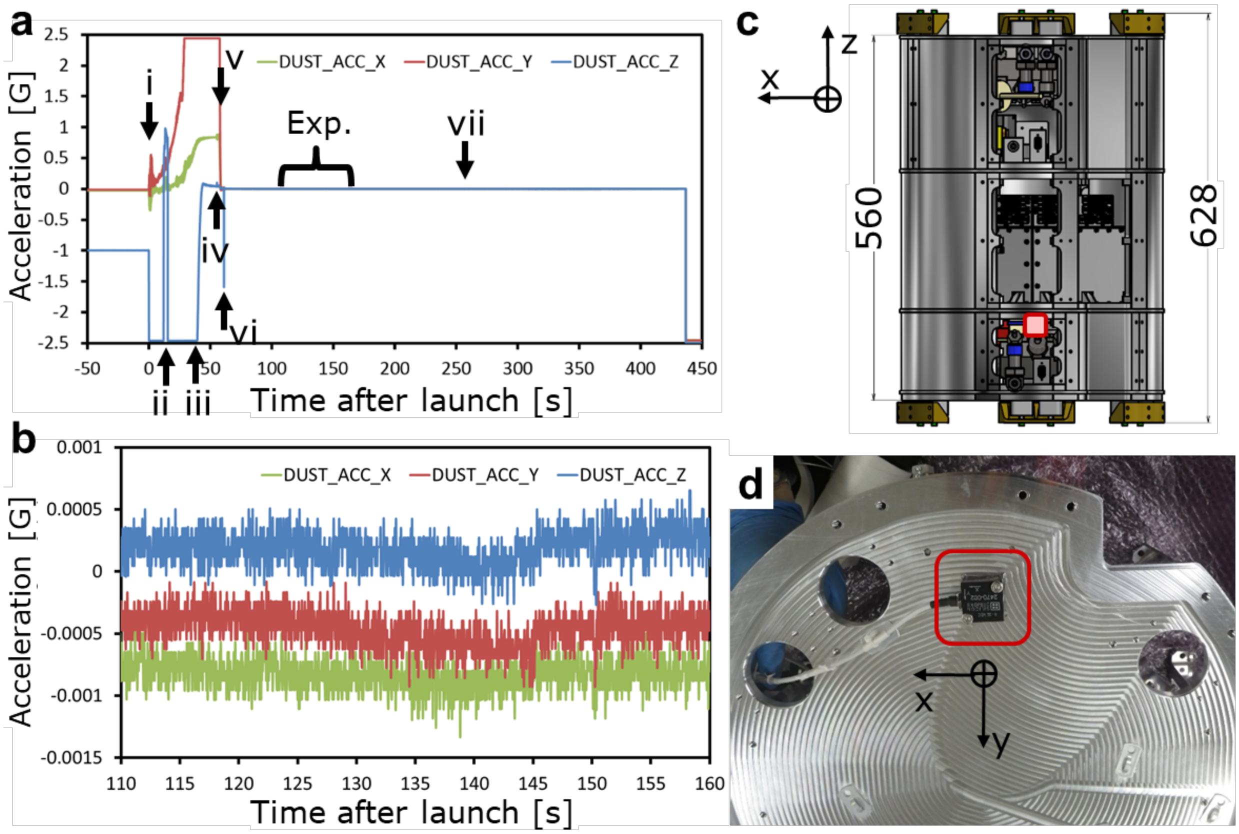

Fig. S1. Acceleration in units of Earth gravity for the sounding rocket during the microgravity experiment. (**a**) Time evolution from 50 s before launch to the end of telemetry. The labels indicate the events of (**i**) launch at 0 s, (**ii**) burn out of the first rocket stage at 13 s, (**iii**) burn out of the second rocket stage at 42 s, (**iv**) nose-tip ejection at 56 s, (**v**) despinning by yo-yo release at 57 s, (**vi**) motor separation at 61 s, and (**vii**) apogee (244.7 km) at 257 s. "Exp." indicates the interval during which the microgravity experiment was run. (**b**) Magnified graph of the acceleration around 0 *g*. The origin of noise is both mechanical and electrical. The origin of the difference in the absolute values (offset) might be caused by the manufacturing process. The gravity levels were all of the order of $<2.5 \times 10^{-4}$ *g* for all three axes during the sounding-rocket flight. (**c**) Schematics of the whole experimental system, which consists of the interferometer (bottom), the interface circuit (center), and the infrared spectrometer (top; not discussed in this paper), connected to bumpers on the top and bottom. The bumpers connect to the inner wall of the rocket skin. The numbers show the scale in millimeters. The red box shows an accelerometer. (**d**). Photograph of the triaxial analog accelerometer module positioned above the nucleation chamber and on the base plate of the experimental system. The direction of the lasers in the chamber corresponds to the *x*-axis of acceleration.

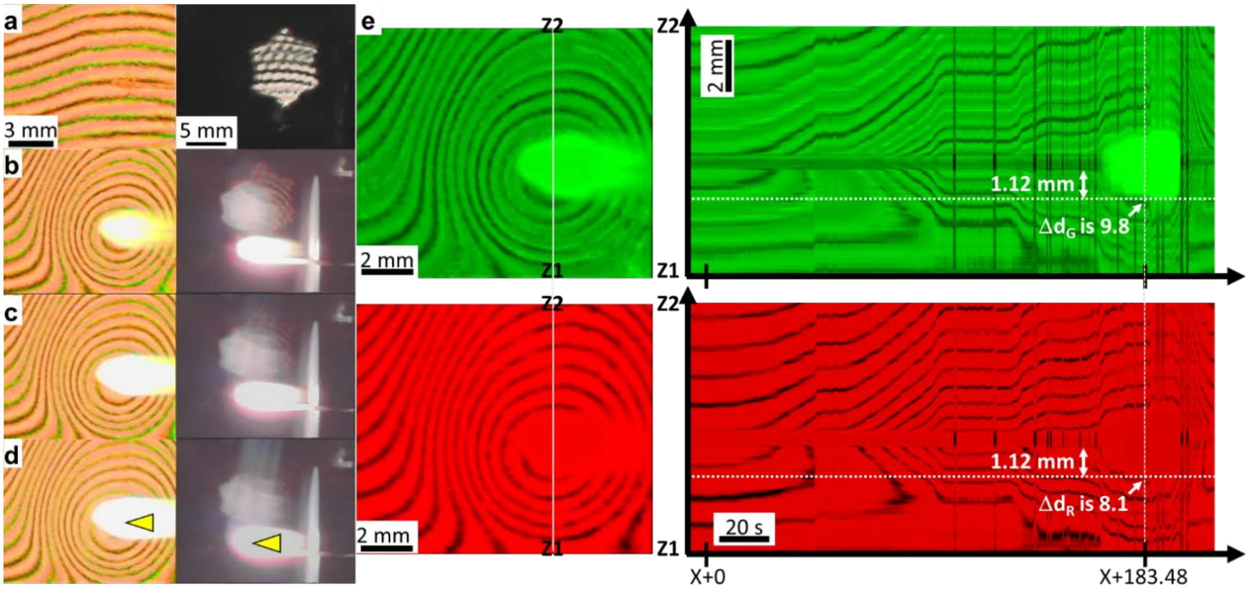

Fig. S2. Snapshots of in-situ observations at the moment of nucleation of titanium in gravity. The left- and right-hand panels show double-wavelength interference fringes and real images, respectively: (**a**) before heating, (**b**) just before nucleation (X + 176.17 s), (**c**) soon after nucleation (X + 177.04 s), and (**d**) clear nucleation (X + 177.51 s), where X is the elapsed time after heating began. The real image was observed through a beam splitter and a viewport of the chamber; a second viewport and beam splitter at the back of the chamber caused the images to overlap and become difficult to distinguish objects. The bright hexagonal star-shaped areas in the real images were caused by stray light reflected from the aperture of the camera lens onto the optics. The scale bar in the real image is correct only at the focused position because objects closer to the camera appear larger and objects farther away appear smaller. (**e**) Color-separated interference fringes. The left-hand snapshot corresponds to **b.** On the right is a time series of images at the position indicated by the vertical line Z1–Z2. The vertical lines and horizontal dotted lines in the time-series images indicate the time of nucleation and the position of nucleation at 1.12 mm from the evaporation source, respectively. The arrow indicates deviations of the interference fringes of Δd_G and Δd_R at the nucleation position for the green and red lasers, respectively. This method of analyzing interference fringes follows our previous works (22, 23).

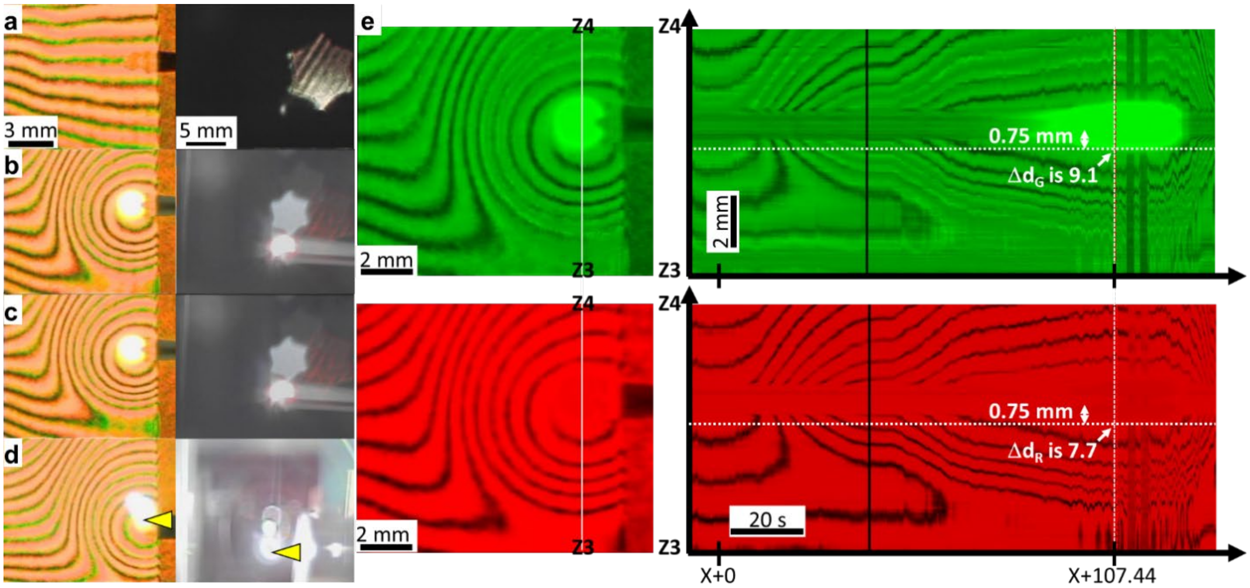

Fig. S3. Snapshots of in-situ observations at the moment of nucleation of carbon in gravity. The left- and right-hand panels show double-wavelength interference fringes and real images, respectively: (**a**) before heating, (**b**) just before nucleation (X + 107.44 s), (**c**) clear nucleation (X + 117.39 s), and (**d**) clear nucleation with a different magnification of the real image, taken during a different run. X is the elapsed time after heating started. The real image was observed through a beam splitter and a viewport of the chamber; a second viewport and beam splitter at the back of the chamber caused the images to overlap and become difficult to distinguish objects. The bright hexagonal star-shaped areas in the real images were caused by stray light reflected from the aperture of the camera lens onto the optics. The scale bar in the real image is correct only at the focused position because objects closer to the camera appear larger and objects farther away appear smaller. (**e**) Color-separated interference fringes. The left-hand snapshot corresponds to **b**. On the right is a time series of images at the position indicated by the vertical line Z3–Z4. The vertical dashed lines and horizontal dotted lines in the time-series images indicate the time of nucleation and the position of nucleation at 0.75 mm from the evaporation source, respectively. The arrow indicates deviations of the interference fringes of Δd_G and Δd_R at the nucleation position for the green and red lasers, respectively. This method of analyzing interference fringes follows our previous works (22, 23).

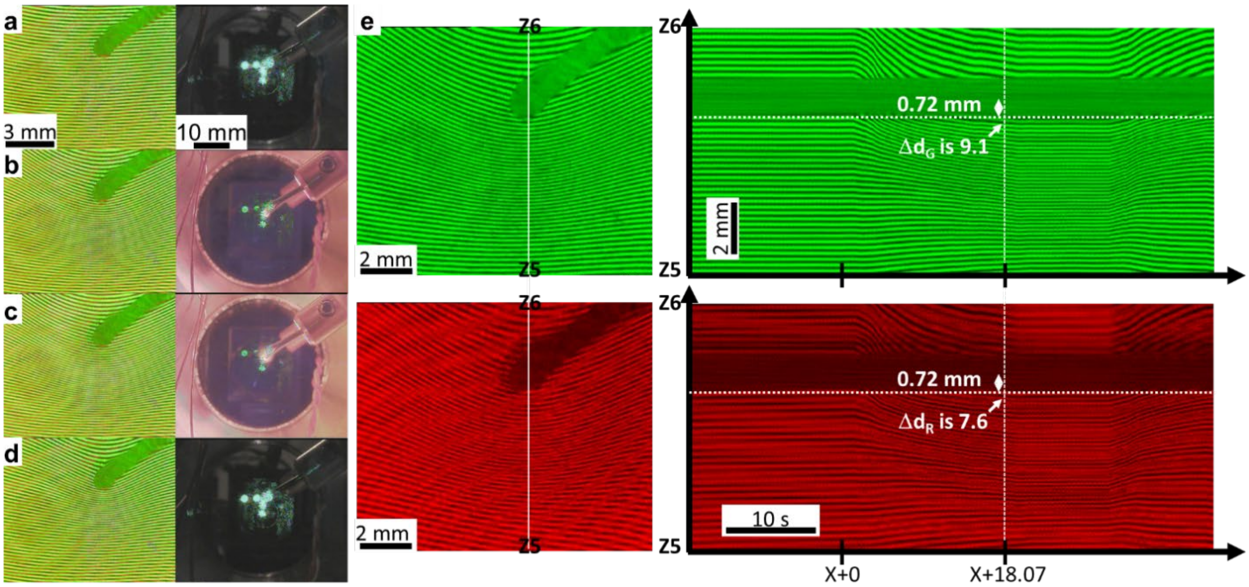

Fig. S4. Snapshots of in-situ observations at the moment of nucleation of TiC in gravity. The left- and right-hand panels show double-wavelength interference fringes and real images, respectively: (**a**) before heating, (**b**) just before nucleation (X + 18.07 s), (**c**) clear nucleation (X + 19.62 s) and (**d**) after heating (X + 34.47 s). X is the elapsed time after heating started. The real image was observed through a beam splitter and a viewport of the chamber; a second viewport and beam splitter at the back of the chamber caused the images to overlap and become difficult to distinguish objects. The scale bar in the real image is correct only at the focused position because objects closer to the camera appear larger and objects farther away appear smaller. (**e**) Color-separated interference fringes. The left-hand snapshot corresponds to **b**. On the right is a time series of images at the position indicated by the vertical line Z5–Z6. The vertical dashed lines and horizontal dotted lines in the time-series images indicate the time of nucleation and the position of nucleation at 0.72 mm from the evaporation source, respectively. The arrow indicates deviations of the interference fringes of Δd_G and Δd_R at the nucleation position for the green and red lasers, respectively. This method of analyzing interference fringes follows our previous works (22, 23).

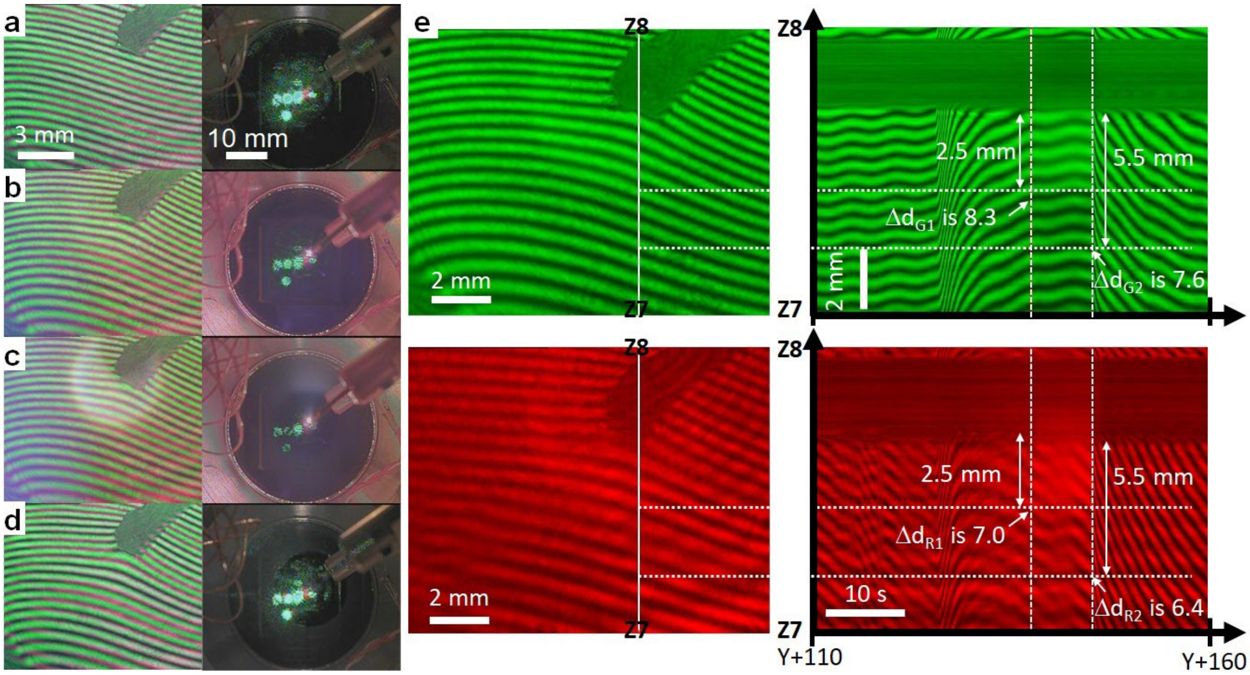

Fig. S5. Snapshots of in-situ observations at the moment of nucleation in the microgravity environment. The left- and right-hand panels show double-wavelength interference fringes and real images, respectively, (**a**) before heating (*Y* + 125.02 s), (**b**) just before nucleation (*Y* + 134.30 s), (**c**) during nucleation (*Y* + 139.87 s) and (**d**) after heating (*Y* + 147.17 s), where *Y* is the time of launch. The real image was observed through a beam splitter and a viewport of the chamber; a second viewport and beam splitter at the back of the chamber caused the images to overlap and become difficult to distinguish objects. The size of the copper electrodes shown at the upper right-hand corner in the real images is 6.0 mm. The bright circular region seen in the interference image **c** is due to the strong light from the evaporation source directly entering the CCD camera. (**e**) Color-separated interference fringes. The left-hand snapshot corresponds to **c.** On the right is a time series of images at the position indicated by a vertical line Z7–Z8. The vertical dashed lines and horizontal dotted lines in the time-series images indicate the time of nucleation and the position of nucleation at 5.5 mm from the evaporation source, respectively. The arrow indicates deviations of the interference fringes of Δd_G and Δd_R at the nucleation position for the green and red lasers, respectively. This method of analyzing interference fringes follows our previous works (22, 23).

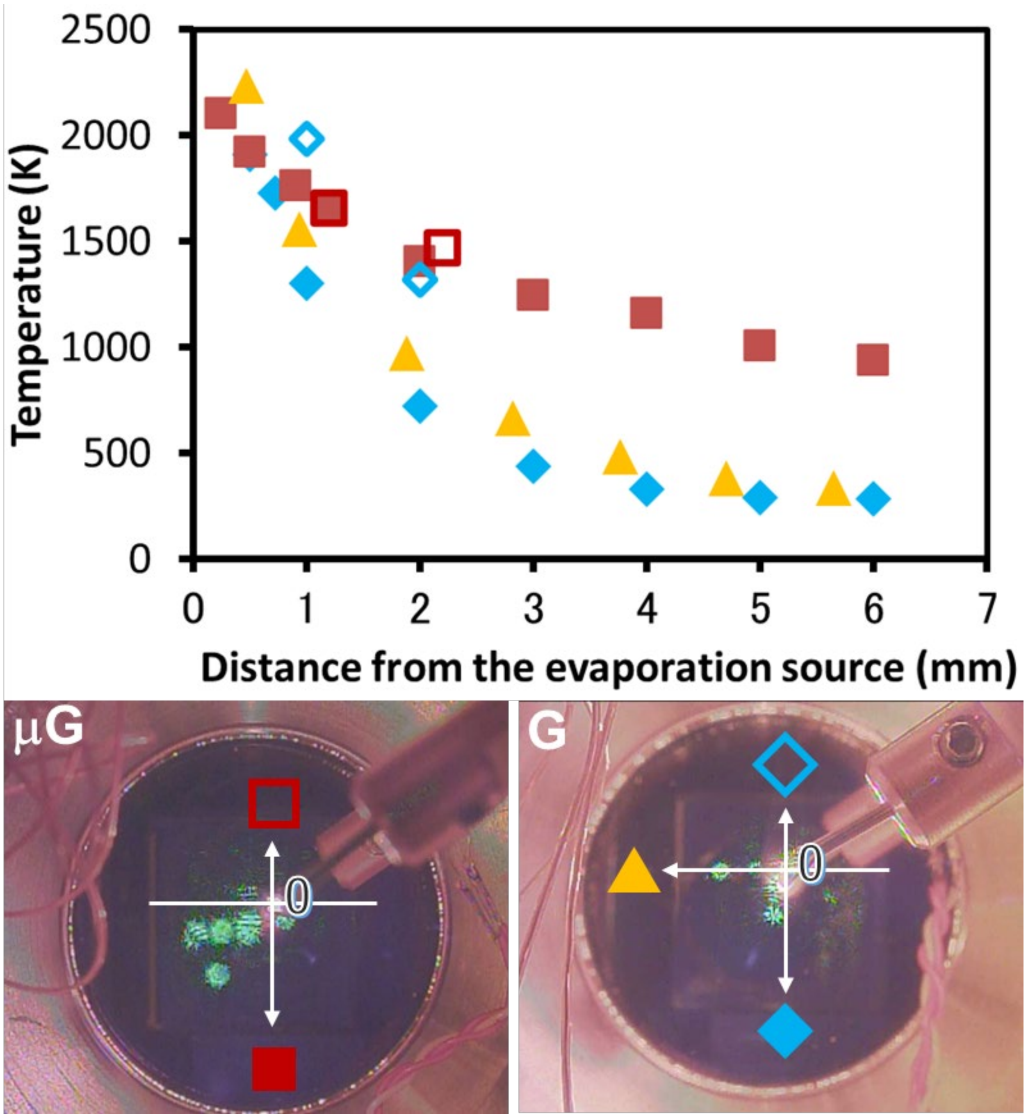

Fig. S6. Comparison of the temperature profiles around the evaporation source in the microgravity and ground experiments. The snapshot in the microgravity experiment was taken immediately before nucleation at an applied voltage of ~12 V. The red square, the yellow triangle, and the blue diamond represent temperatures in the *z*-direction of the acceleration in the sounding-rocket experiment, the horizontal direction in gravity, and the gravitational direction, respectively.

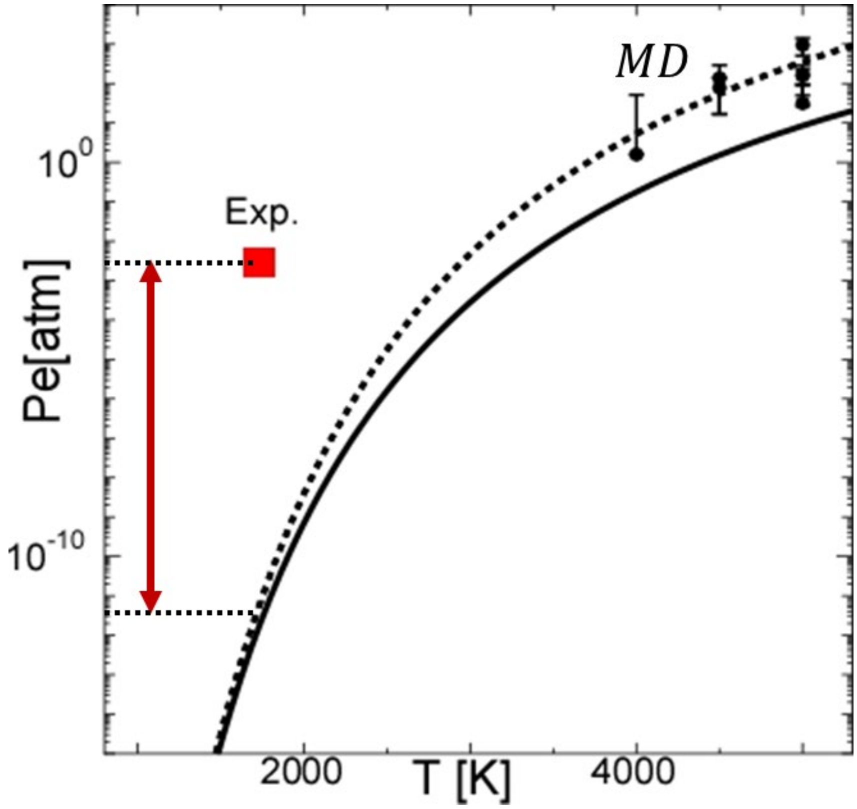

Fig. S7. Equilibrium vapor pressure of TiC. The solid line is taken from Ref. 57; Log *P* [atm] = 7.652 – 33600/*T*. The black circles and the dotted line were determined from a molecular-dynamics simulation of a liquid slab in equilibrium with the vapor phase (59). The simulations were performed with the Large-Scale Atomic/Molecular Massively Parallel Simulator (LAMMPS; 60) by using modified embedded-atom method (MEAM) interatomic potentials (61) for the Ti–C systems and a constant number of molecules (3000), volume, and temperature ensemble with a periodic boundary condition. The molecular dynamics simulations were performed at three temperatures: 4000 K, 4500 K, and 5000 K. At temperatures below 4000 K, the equilibrium vapor pressure is small and cannot be calculated without a large error. The red square shows the temperature and partial pressure of TiC just before the nucleation event shown in Fig. S4. The double-headed arrow shows the difference between the partial pressure and the equilibrium vapor pressure of TiC at the nucleation temperature (~1700 K), i.e., the supersaturation ratio.

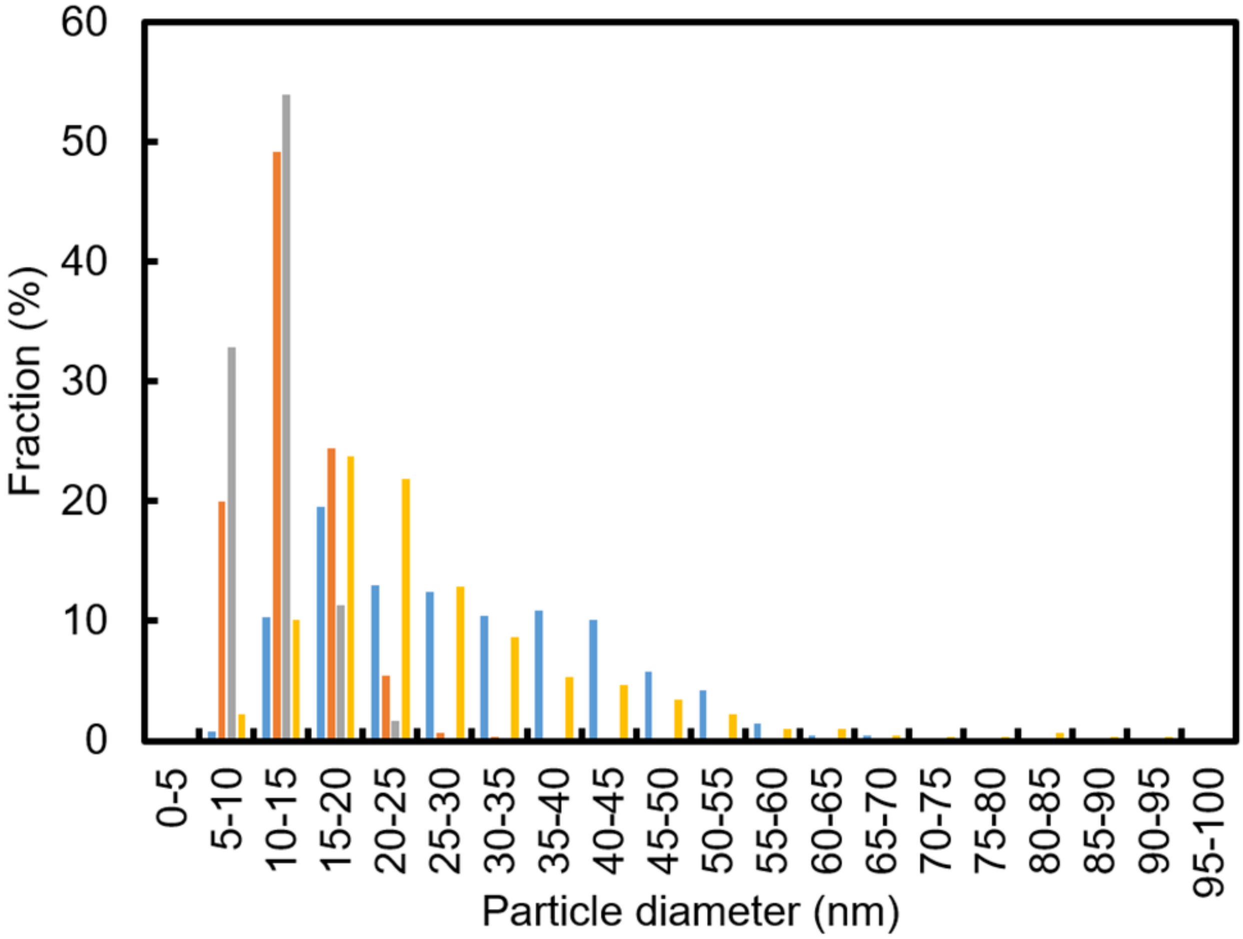

Figure S8. **Fractional size distribution of the experimentally produced particles shown in Fig. 2.** The diameters of the particles were measured manually. Cases in which two particles overlapped or were connected by a thick neck and were difficult to measure unambiguously are omitted. Fine particles are not included in this distribution because they invariably overlapped with larger particles. The bars from left to right correspond to Figs. 3a–d, respectively; i.e., Ti particles formed in gravity (blue), carbon particles formed in gravity (orange), TiC particles formed in gravity (gray), and particles produced in microgravity (yellow). The total numbers for each of these particles were 1637, 1078, 1553 and 1458, respectively.

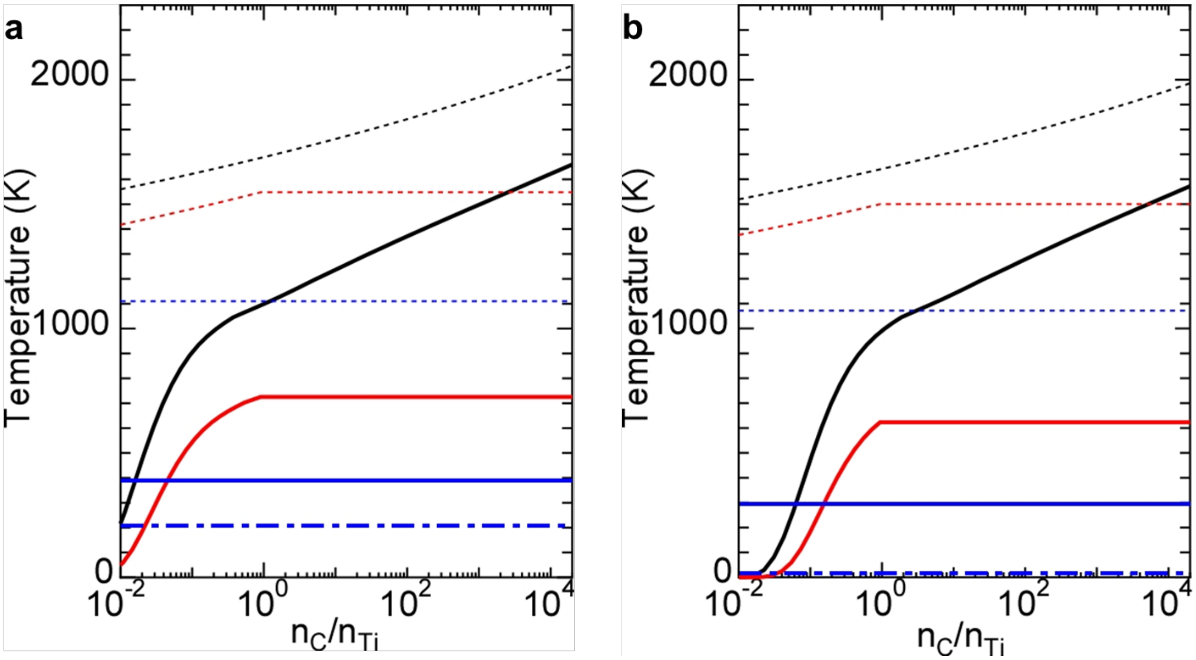

Figure S9. Dependence of the nucleation temperature on the number density. The plots show the nucleation temperature in a gas outflow from a Type II supernova, calculated by using the values of α and σ obtained from the gravitational experiment (solid lines) in Figs. 4a–c and from the microgravity experiment (dot/dash line) in Fig. 4d in terms of the C/Ti ratio, which depends, in turn, on the C/O ratio, with a cooling time of one year. The dotted lines show the equilibrium temperature for Ti (blue), C (black), and TiC (red) in terms of the C/Ti ratio. The gaseous number density ($\approx 3.6 \times 10^{17}$ m^{-3}) (47) was fixed, as in Fig. 4e, and number density of Ti was changed from 9.2×10^{10} m^{-3} in Fig. 4e to 4.6×10^{10} m^{-3} in **a** and 9.2×10^{9} m^{-3} in **b**.

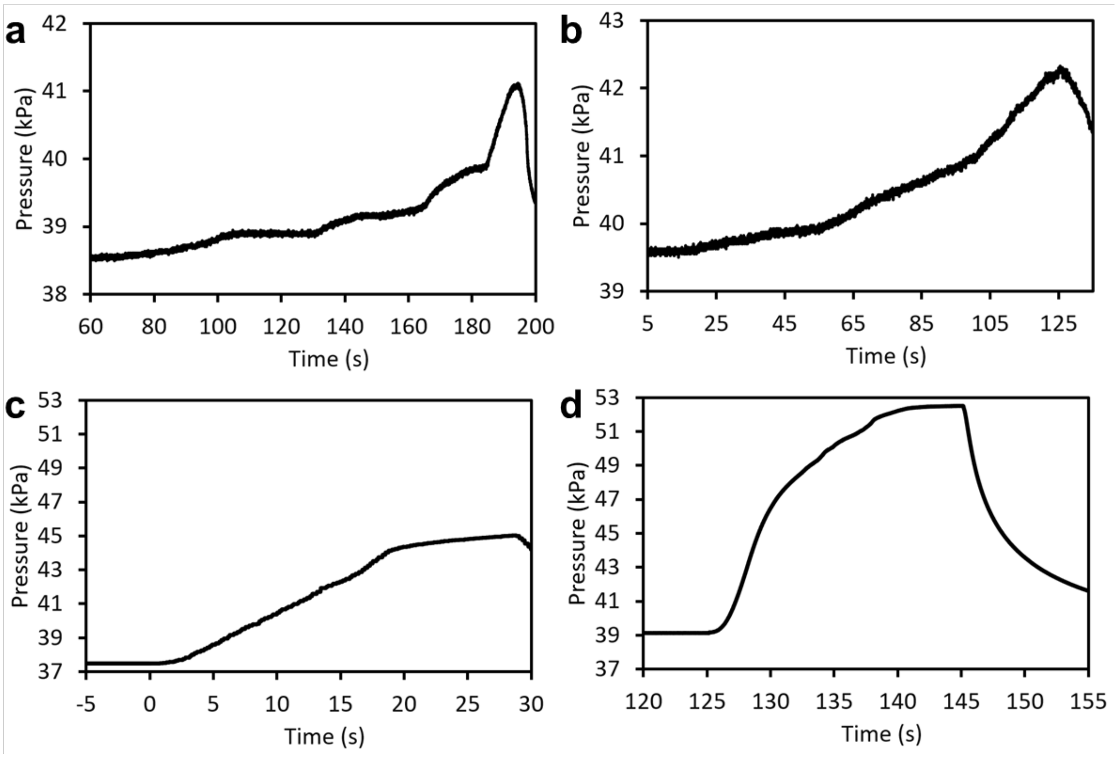

Figure S10. **Total pressure variations with time. a–c**. Experiments with Ti, C, and TiC particles, respectively, in gravity. **d**. Microgravity experiment. The times in a–d correspond to those in Figs. S2–S4, respectively. The evaporation source was heated manually in the ground experiment, whereas in the microgravity experiment, the voltage was raised to a constant value in the program. Consequently, the heating time was different in each experiment. The total pressure increase in the ground experiment was smaller because of the larger chamber size (21). Differences in both the pressure and heating time had a small effect on nucleation, e.g., the difference in the collision frequency was less than 20%.

REFERENCES AND NOTES

1. N. J. Woolf, E. P. Ney, Circumstellar infrared emission from cool stars. *Astrophys. J.* **155**, L181–L184 (1969).

2. T. T. Takeuchi, V. Buat, D. Burgarella, The evolution of the ultraviolet and infrared luminosity densities in the Universe at $0 < z < 1$. *Astron. Astrophys.* **440**, L17−L20 (2005).

3. W. A. Schutte, A. G. G. M. Tielens, Theoretical studies of the infrared emission from circumstellar dust shells: The infrared characteristics of circumstellar silicates and the mass-loss rate of oxygen-rich late-type giants. *Astrophys. J.* **343**, 369–392 (1989).

4. T. Hama, N. Watanabe, Surface processes on interstellar amorphous solid water: Adsorption, diffusion, tunneling reactions, and nuclear-spin conversion. *Chem. Rev.* **113**, 8783–8839 (2013).

5. J. A. Nuth III, N. M. Johnson, Complex protostellar chemistry. *Science* **336**, 424–425 (2012).

6. A. J. Weinberger, Planetary science: Construction-site inspection. *Nature* **433**, 114–115 (2005).

7. E. Anders, E. K. Zinner, Interstellar grains in primitive meteorites: Diamond, silicon carbide, and graphite. *Meteoritics* **28**, 490–514 (1993).

8. U. Ott, Interstellar grains in meteorites. *Nature* **364**, 25–33 (1993).

9. L. R. Nittler, C. M. O.'D. Alexander, X. Gao, R. M. Walker, E. K. Zinner, Interstellar oxide grains from the Tieschitz ordinary chondrite. *Nature* **370**, 443–446 (1994).

10. T. J. Bernatowicz, S. Amari, E. K. Zinner, R. S. Lewis, Interstellar grains within interstellar grains. *Astrophys. J.* **373**, L73–L76 (1991).

11. T. J. Bernatowicz, R. Cowsik, P. C. Gibbons, K. Lodders, B. Fegley, S. Amari, R. S. Lewis, Constraints on stellar grain formation from presolar graphite in the Murchison meteorite. *Astrophys. J.* **472**, 760–782 (1996).

12. T. K. Croat, T. Bernatowicz, S. Amari, S. Messenger, F. J. Stadermann, Structural, chemical, and isotopic microanalytical investigations of graphite from supernovae. *Geochim. Cosmochim. Acta* **67**, 4705–4725 (2003).

13. T. K. Croat, F. J. Stadermann, T. J. Bernatowicz, Presolar graphite from AGB stars: Microstructure and *s*-process enrichment. *Astrophys. J.* **631**, 976–987 (2005).

14. F. J. Stadermann, T. K. Croat, T. J. Bernatowicz, S. Amari, S. Messenger, R. M. Walker, E. Zinner, Supernova graphite in the NanoSIMS: Carbon, oxygen and titanium isotopic compositions of a spherule and its TiC sub-components. *Geochim. Cosmochim. Acta* **69**, 177–188 (2005).

15. K. Lodders, B. Fegley Jr., The origin of circumstellar silicon carbide grains found in meteorites. *Meteoritics* **30**, 661–678 (1995).

16. C. M. Sharp, G. J. Wasserburg, Molecular equilibria and condensation temperatures in carbon-rich gases. *Geochim. Cosmochim. Acta* **59**, 1633–1652 (1995).

17. T. Chigai, T. Yamamoto, T. Kozasa, Formation conditions of presolar TiC core–graphite mantle spherules in the Murchison meteorite. *Astrophys. J.* **510**, 999–1010 (1999).

18. T. Chigai, T. Yamamoto, T. Kozasa, Heterogeneous condensation of presolar titanium carbide core–graphite mantle spherules. *Meteorit. Planet. Sci.* **37**, 1937–1951 (2002).

19. K. Lodders, They came from the deep in the supernova: The origin of TiC and metal subgrains in presolar graphite grains. *Astrophys. J.* **647**, L37–L40 (2006).

20. M. Bose, C. Floss, F. J. Stadermann, An investigation into the origin of Fe-rich presolar silicates in Acfer 094. *Astrophys. J.* **714**, 1624–1636 (2010).

21. Y. Kimura, K. K. Tanaka, H. Miura, L. Tsukamoto, Direct observation of the homogeneous nucleation of manganese in the vapor phase and determination of surface free energy and sticking coefficient. *Cryst. Growth Des.* **12**, 3278–3284 2012.

22. Y. Kimura, K. K. Tanaka, T. Nozawa, S. Takeuchi, Y. Inatomi, Pure iron grains are rare in the Universe. *Sci. Adv.* **3**, 1601992 (2017).

23. Y. Kimura, K. K. Tanaka, Y. Inatomi, F. T. Ferguson, J. A. Nuth III, Inefficient growth of SiOx grains: Implications for circumstellar outflows. *Astrophys. J.* **934**, L10 (2022).

24. R. Becker, W. Döring, Kinetische behandlung der keimbildung in übersättigten dämpfen. *Ann. Phys.* **416**, 719–752 (1935).

25. J. B. Zeldovich, On the theory of formation of a new phase,cavitation. *J. Exp. Theor. Phys.* **12**, 525–536 (1942).

26. J. Feder, K. C. Russel, J. Lothe, G. M. Pound, Homogeneous nucleation and growth of droplets in vapours. *Adv. Phys.* **15**, 111–178 (1966).

27. K. K. Tanaka, H. Tanaka, K. Nakazawa, Non-equilibrium condensation in a primordial solar nebula: Formation of refractory metal nuggets. *Icarus* **160**, 197–207 (2002).

28. A. Sarangi, I. Cherchneff, Condensation of dust in the ejecta of Type II-P supernovae. *Astron. Astrophys.* **575**, A95 (2015).

29. I. Cherchneff, Dust formation in carbon-rich Wolf-Rayet colliding winds, in *International Workshop on Wolf-Rayet Stars: Proceedings of an International Workshop*, W.-R. Hamann, A. Sander, H. Todt, Eds. (Potsdam Univ. Press, 2015), pp. 269–274; https://publishup.uni-potsdam.de/opus4-ubp/frontdoor/deliver/index/docId/8426/file/wr_stars_proceedings.pdf.

30. Y. Kimura, K. Tsukamoto, Homogeneous nucleation of smoke particles and its relationship with cosmic dust particles, in *New Perspectives on Mineral Nucleation and Growth*, A. E. S. Van Driessche, M. Kellermeier, L. G. Benning, D. Gebauer, Eds. (Springer International, 2017), pp. 339–351.

31. Y. Kimura, H. Miura, K. Tsukamoto, C. Li, T. Maki, Interferometric in situ observation during nucleation and growth of WO_3 nanocrystals in vapor phase. *J. Cryst. Growth* **316**, 196–200 (2011).

32. S. Ishizuka, Y. Kimura, I. Sakon, H. Kimura, T. Yamazaki, S. Takeuchi, Y. Inatomi, Sounding-rocket microgravity experiments on alumina dust. *Nat. Commun.* **9**, 3820 (2018).

33. T. Yamamoto, H. Hasegawa, Grain formation through nucleation process in astrophysical environment. *Prog. Theor. Phys.* **58**, 816–828 (1977).

34. T. Nozawa, T. Kozasa, Formulation of non-steady-state dust formation process in astrophysical environments. *Astrophys. J.* **776**, 24 (2013).

35. K. K. Tanaka, A. Kawano, H. Tanaka, Molecular dynamics simulations of the nucleation of water: Determining the sticking probability and formation energy of a cluster, *J. Chem. Phys.* **140**, 114302 (2014).

36. K. Zhou, H. P. Wang, J. Chang, B. Wei, Experimental study of surface tension, specific heat and thermal diffusivity of liquid and solid titanium. *Chem. Phys. Lett.* **639**, 105–108 (2015).

37. W. Li, L.-h. Fang, J.-h. Gong, First-principles study of TiC(110) surface. *Trans. Nonferrous Met. Soc. China* **22**, 170–174 (2012).

38. R. G. Tabak, J. P. Hirth, G. Mevrick, T. P. Roark, The nucleation and expulsion of carbon particles formed in stellar atmospheres. *Astrophys. J.* **196**, 457–463 (1975).

39. H.-P. Gail, R. Keller, E. Sedlmayr, Dust formation in stellar winds. I. A rapid computational method and application to graphite condensation. *Astron. Astrophys.* **133**, 320–332 (1984).

40. H. Zhang, B. Chen, J. F. Banfield, The size dependence of the surface free energy of titania nanocrystals. *Phys. Chem. Chem. Phys.* **11**, 2553–2558 (2009).

41. Y. Kimura, Phenomena of nanoparticles in relation to the solar system, in *Nanodust in the Solar System: Discoveries and Interpretations*, I. Mann, N. Meyer-Vernet, A. Czechowski, Eds. (Springer, 2012), pp. 31–46.

42. S. Ishizuka, Y. Kimura, S. Yokoi, T. Yamazaki, R. Sato, T. Hama, Self-assembly of MoO_3 needles in gas current for cubic formation pathway. *Nanoscale* **9**, 10109–10116 (2017).

43. D. Li, M. H. Nielsen, J. R. I. Lee, C. Frandsen, J. F. Banfield, J. J. De Yoreo, Direction-specific interactions control crystal growth by oriented attachment. *Science* **336**, 1014–1018 (2012).

44. J. J. De Yoreo, P. U. P. A. Gilbert, N. A. J. M. Sommerdijk, R. L. Penn, S. Whitelam, D. Joester, H. Zhang, J. D. Rimer, A. Navrotsky, J. F. Banfield, A. F. Wallace, F. M. Michel, F. C. Meldrum, H. Cölfen, P. M. Dove, Crystallization by particle attachment in synthetic, biogenic, and geologic environments. *Science* **349**, 6760 (2015).

45. P. G. Vekilov, Crystallization tracked atom by atom. *Nature* **570**, 450–452 (2019).

46. S. Yatsuya, A. Yanagida, K. Yamauchi, K. Mihama, Attempt to form ultrafine particles with hydride and amorphous structure. *J. Cryst. Growth* **70**, 536–540 (1984).

47. T. Rauscher, A. Heger, R. D. Hoffman, S. E. Woosley, Nucleosynthesis in massive stars with improved nuclear and stellar physics. *Astrophys. J.* **576**, 323–348 (2002).

48. T. C. Wallace Sr., D. P. Butt, Review of diffusion and vaporization of Group 4 and 5 transition metal carbides, in *The Chemistry of Transition Metal Carbides and Nitrides*, S. T. Oyama, Ed. (Blackie, 1996), pp. 53–90.

49. H. Mori, H. Yasuda, T. Kamino, High-resolution electron microscopy study of spontaneous alloying in gold clusters. *Philos. Mag. Lett.* **69**, 279–283 (1994).

50. A. Bideau-Mehu, Y. Guern, R. Abjean, A. Johannin-Gilles, Measurement of refractive indices of neon, argon, krypton and xenon in the 253.7–140.4 nm wavelength range. Dispersion relations and estimated oscillator strengths of the resonance lines. *J. Quant. Spectrosc. Radiat. Transfer* **25**, 395–402 (1981).

51. A. Börzsönyi, Z. Heiner, M. P. Kalashnikov, A. P. Kovács, K. Osvay, Dispersion measurement of inert gases and gas mixtures at 800 nm. *Appl. Optics* **47**, 4856–4863 (2008).

52. E. R. Peck, D. J. Fisher, Dispersion of argon, *J. Opt. Soc. Am.* **54**, 1362–1364 (1964).

53. Y. Clergent, C. Durou, M. Laurens, Refractive index variations for argon, nitrogen, and carbon dioxide at $\lambda = 632.8$ nm (He−Ne laser light) in the range $288.15\ \mathrm{K} \le T \le 323.15\ \mathrm{K}$, $0 < p < 110$ kPa. *J. Chem. Eng. Data* **44**, 197–199 (1999).

54. P. B. Johnson, R. W. Christy, Optical constants of transition metals: Ti, V, Cr, Mn, Fe, Co, Ni, and Pd. *Phys. Rev. B* **9**, 5056–5070 (1974).

55. J. Pflüger, J. Fink, W. Weber, K. P. Bohnen, G. Crecelius, Dielectric properties of TiC_x, TiN_x, VC_x, and VN_x from 1.5 to 40 eV determined by electron-energy-loss spectroscopy. *Phys. Rev. B* **30**, 1155–1163 (1984).

56. A. B. Djurišić, E. H. Li, Optical properties of graphite. *J. Appl. Phys.* **85**, 7404–7410 (1999).

57. *CRC Handbook of Chemistry and Physics,* D. R. Lide, Ed. (CRC Press, ed. 76, 1996), section 6.

58. *The Refractory Carbides*, E. K. Storms, Ed. (Academic Press, 1967).

59. R. Sakamaki, A. K. Sum, T. Narumi, K. Yasuoka, Molecular dynamics simulations of vapor/liquid coexistence using the nonpolarizable water models. *J. Chem. Phys.* **134**, 124708 (2011).

60. S. Plimpton, Fast parallel algorithms for short-range molecular dynamics. *J. Comput. Phys.* **117**, 1–19 (1995).

61. Y.-M. Kim, B.-J. Lee, Modified embedded-atom method interatomic potentials for the Ti-C and Ti-N binary systems. *Acta Mater.* **56**, 3481–3489 (2008).
